# Adjuvant nutritional support for patients with acute-on-chronic liver failure reduce the risk of clinical complications

**DOI:** 10.3389/fnut.2025.1734215

**Published:** 2026-01-09

**Authors:** Wenjing Ma, Guoyuan Qiu, Hong Liu

**Affiliations:** 1 Interventional Therapy Center, West China Hospital, Sichuan University, Chengdu, Sichuan, China; 2 Interventional Ward, West China Hospital, Sichuan University, Chengdu, Sichuan, China; 3 West China Hospital, Sichuan University, Chengdu, Sichuan, China; 4 West China School of Nursing, Sichuan University, Chengdu, Sichuan, China; 5 Division of Gastrointestinal Surgery, Department of General Surgery, West China Hospital, Sichuan University, Chengdu, Sichuan, China; 6 Department of Clinical Nutrition, West China Hospital, Sichuan University, Chengdu, Sichuan, China

**Keywords:** acute-on-chronic liver failure, complication, NRS 2002, nutrition risk assessment, nutritional intervention

## Abstract

**Background:**

Acute-on-chronic liver failure (ACLF) patients face high risks of nutritional risk and clinical complications, but whether adjuvant nutritional support reduces adverse complications remains unclear.

**Methods:**

This study included 6,097 hospitalized ACLF patients at West China Hospital. The Nutrition Risk Screening (NRS 2002) was used to assess the nutritional risk of the patients. Additional personalized enteral or parenteral nutrition was provided. The Kruskal–Wallis test and chi-square test compared continuous and categorical variables. Multivariate logistic regression analyzed odds ratios (ORs) and 95% confidence intervals (95% CI) for nutritional intervention and adverse outcomes, while the Cox model evaluated all-cause mortality risk.

**Results:**

Among participants (median age 53 years, 69.2% male), 52.5% (3,201) had nutritional risk, who were older, had lower body mass index (BMI), longer hospital stays, and higher rates of infection, hepatorenal syndrome, hepatic encephalopathy, coagulation disorders, and mortality (*p* < 0.01). After confounding adjustment, nutritional intervention in high-risk patients was associated with lower frequency of ascites (OR = 0.50, 95% CI: 0.31–0.80), infection (OR = 0.70, 95% CI: 0.52–0.94), and spontaneous bacterial peritonitis (SBP) (OR = 0.34, 95% CI: 0.24–0.49). Moreover, for patients without nutritional risk, nutritional intervention was also associated with lower frequency of ascites [0.26 (0.12–0.55)], SBP [0.30 (0.17–0.54)], and coagulation disorder [0.35 (0.17–0.72)]. Subgroup analysis showed consistent conclusions in most subgroups. However, nutritional intervention had no significant effect on improving all-cause mortality.

**Conclusion:**

Over half of liver failure patients have nutritional risk. Adjuvant nutritional support was associated with significantly lower frequencies of clinical complications, especially ascites and SBP, regardless of nutritional risk.

## Introduction

Acute-on-chronic liver failure (ACLF), characterized by decompensation of liver function, is one of the most urgent end stage liver diseases (1, 2). Patients with ACLF often suffer from a series of severe complications (3). These complications not only severely affect the quality of life of patients but also significantly increase the mortality rate.

The functions of synthesis, metabolism, and detoxification are severely impaired in the state of liver failure. Meanwhile, patients often experience digestive symptoms such as loss of appetite, nausea, and vomiting, which further affect the intake and absorption of nutrients. Thus, patients with ACLF generally face a high nutritional risk (4–6). Malnutrition is a common complication in patients with ACLF (7), and malnutrition is seen in up to 90% of patients with cirrhosis (8). However, nutrition has a central prognostic and therapeutic role in the management of patients with liver disease. Studies revealed that approximately 50% of outpatients with alcohol-related liver disease (ALD) suffer from malnutrition, while nearly all inpatients with ALD are affected. Notably, malnutrition has a negative impact on treatment responses and patient outcomes (9, 10). Individualized nutritional interventions may improve prognosis of patients with ACLF (11).

At present, many guidelines recommend nutritional support for patients with liver diseases (12, 13). However, most of them focus on patients with chronic liver disease, moreover, there is insufficient data to demonstrate that nutritional support can reduce the risk of complications. Therefore, to explore nutritional support therapy for ACLF patients, we retrospectively collected inpatients diagnosed with ACLF in our hospital and explored whether adjuvant nutritional support will reduce the risk of clinical complications and all-cause mortality.

## Methods

### Patients and research design

This study retrospectively enrolled patients diagnosed with ACLF according to the criteria of the Asian Pacific Association for the Study of the Liver (APASL) and treated at West China Hospital during 2018–2023. All patients undergone nutritional risk assessment by nurses upon admission and received a standardized medical management during hospitalization. Notably, according to international guidelines (12–14), nutritional support is recommended as standard supportive care for liver failure. In this study, patients who did not receive nutritional intervention (*n* = 2,896) were a highly selected subgroup, with enrollment criteria restricted to three scenarios: (1) Very mild ACLF (Child–Pugh A grade, no overt complications, and autonomous oral intake meeting ≥80% of daily energy requirements); (2) Terminal-stage ACLF (Child–Pugh C grade with multi-organ failure, where nutritional support was deemed clinically futile by the multidisciplinary team); (3) Patient/family refusal of nutritional intervention after full informed consent. To mitigate inherent selection bias, we adjusted for disease severity in multivariate logistic regression model. Additionally, subgroup analyses stratified by ACLF severity were performed to validate the robustness of associations between nutritional support and complications.

### Nutritional risk assessment

The Nutritional Risk Screening-2002 (NRS-2002) was used to screen for nutritional risk in patients with liver failure. The NRS-2002 screening tool, which has been validated in liver failure populations, is designed to identify individuals at nutritional risk (score ≥3) who may potentially benefit from nutrition intervention to optimize clinical outcomes (15, 16). We selected the NRS-2002 for nutritional risk screening based on its proven applicability in ACLF populations and advantages over liver-specific tools. First, the NRS-2002 can be completed within 24 h of admission without complex auxiliary examinations, adapting to the acute clinical scenario of ACLF (16). Second, unlike RFH-NPT (primarily validated in alcoholic cirrhosis) and LDUST (with limited ACLF data), the NRS-2002 has been validated in HBV-related, alcoholic, and cryptogenic ACLF subgroups (12, 17). Third, it effectively predicts nutritional support responsiveness and clinical outcomes in ACLF, as demonstrated by RCT-derived evidence (16) and ACLF-specific studies (11, 17). These data support its suitability for our heterogeneous ACLF cohort. Malnutrition was defined based on the NRS-2002 criteria (score ≥3) combined with objective indicators: serum albumin <35 g/L, unintentional weight loss >5% in 3 months, or reduced food intake <50% of recommended daily energy for >3 days. Nutritional intervention was initiated if patients met either: (1) NRS-2002 score ≥3 (nutritional risk) plus one objective malnutrition indicator; or (2) NRS-2002 score 1–2 (low nutritional risk) but with persistent food intake <60% of target for >5 days. Patients with NRS-2002 score <3 and stable food intake (≥80% of target) were not recommended for intervention, except for those with physician-documented high risk of malnutrition progression. For patients receiving nutritional support, dietitians customize daily personalized nutritional support plans based on their medical conditions to ensure that their daily energy requirements are met. The personalized nutritional support plan was defined as a tailored strategy integrating multi-dimensional assessments (clinical severity, metabolic status, gastrointestinal tolerance, nutrient absorption) to optimize nutrient delivery, with real-time adjustments based on disease progression. Dietitians conducted baseline and weekly assessments of metabolic markers (serum albumin, prealbumin, glucose, triglycerides) and nutrient levels (fat-soluble vitamins, zinc, selenium) to individualize energy and nutrient targets.

### Nutrition intake

Dietitians conducted detailed nutritional assessments for ACLF patients, with a focus on disease severity [ACLF grade 1/2/3 per APASL criteria (2)] and organ failure status (hepatic, renal, coagulation, encephalopathy, circulatory). Energy requirements were set at 25–30 kcal/kg/day for ACLF Grade 1/2 and 20–25 kcal/kg/day for Grade 3, with a 10–15% increase for patients with hypermetabolism (12, 18). Macronutrient ratios were customized: protein intake ranged from 1.0–1.5 g/kg/day (35–40% as BCAAs for hepatic encephalopathy), carbohydrates accounted for 50–60% of total energy (slow-release formulations preferred), and fat (25–35% of total energy) included medium-chain triglycerides (MCTs) to enhance absorption (12). Special nutrients (glutamine, vitamin D, zinc, selenium) were supplemented based on deficiency status (18, 19). For ACLF grade 1 patients, oral diet was prioritized, with oral nutritional supplements (ONS) containing branched-chain amino acids (BCAAs, 0.8–1.0 g/kg/day) added if oral intake met <60% of basal metabolic rate (BMR). For ACLF grade 2 patients, enteral nutrition (EN) via nasogastric tube was initiated within 48 h of admission, with a stepwise increase in BCAA dosage (1.0–1.2 g/kg/day) and routine supplementation of fat-soluble vitamins (A/D/E/K) and trace elements (zinc, selenium) to address malabsorption caused by hepatic dysfunction. For ACLF grade 3 patients, parenteral nutrition (PN) was combined with EN (if gastrointestinal tolerance allowed) to achieve a protein intake of 1.2–1.5 g/kg/day; glucose-lipid ratio was adjusted to 60:40 to reduce hepatic glucose overload, and glutamine (0.3 g/kg/day) was added to maintain intestinal barrier integrity and inhibit bacterial translocation. Daily gastrointestinal tolerance monitoring (gastric residual volume, stool frequency, symptoms) guided adjustments to infusion rate or composition. EN infusion was reduced by 20% for gastric residual volume >500 mL/6 h, and carbohydrate proportion was adjusted for hyperglycemia (18).

BMR was measured using the InBody 770 device (Biospace, Seoul, South Korea), which employs bioelectrical impedance analysis (BIA) with the following standard conditions: morning fasting state (≥8 h), no strenuous exercise in the previous 24 h, no edema or ascites (or ascites volume <1,000 mL, confirmed by abdominal ultrasound), and measurement performed in a temperature-controlled room (22–25 °C). BMR was calculated via the device’s built-in formula for Asian adults: BMR (kcal/day) = 88.362 + (13.397 × weight/kg) + (4.799 × height/cm) − (5.677 × age/years) for males; BMR = 447.593 + (9.247 × weight/kg) + (3.098 × height/cm) − (4.330 × age/years) for females. BMR was set at 25–30 kcal/kg/day for ACLF grade 1/2 and 20–25 kcal/kg/day for grade 3 (to avoid excessive metabolic burden) with daily energy requirements. Dietitians performed daily follow-ups to monitor gastrointestinal tolerance and adjust nutrient delivery: for patients with hepatic encephalopathy, BCAA proportions were increased to 35–40% of total amino acids to improve ammonia metabolism (12); for those with renal failure, non-essential amino acids were reduced to prevent uremic toxin accumulation. All nutritional formulations avoided excess sodium (<2 g/day) to mitigate ascites progression, consistent with ACLF-specific management principles (14).

### Statistical analysis

For continuous variables, when the data followed a normal distribution, they were presented as the mean and standard deviation (SD). In the case of non-normally distributed data, the median and interquartile range (IQR) were used for presentation. The Lilliefors test was employed to conduct normality testing. Categorical variables were described by presenting their percentages to show the distribution. To compare differences among groups, for continuous variables, the Kruskal–Wallis test was utilized, and for categorical variables, the chi-square test was applied. The logistic regression model was used to calculate the odds ratios (OR) and 95% confidence intervals (CI) to explore the relationship between nutritional support and the risk of clinical complications. Meanwhile, subgroups analyses were performed according to age, gender, body mass index (BMI), diabetes, hypertension, smoking, drinking, Child–Pugh score and cirrhosis status. To further investigate any possible correlations between these subgroups and complication risk, interaction studies were also performed. Cox proportional hazards models were used to assess the association between nutritional support and all-cause mortality. All statistical analyses were carried out using R software together with Zstats v1.0.^1^ A two-sided *p*-value less than 0.05 was defined as statistically significant.

## Results

### Patient enrollment and baseline characteristics

**Figure 1:**
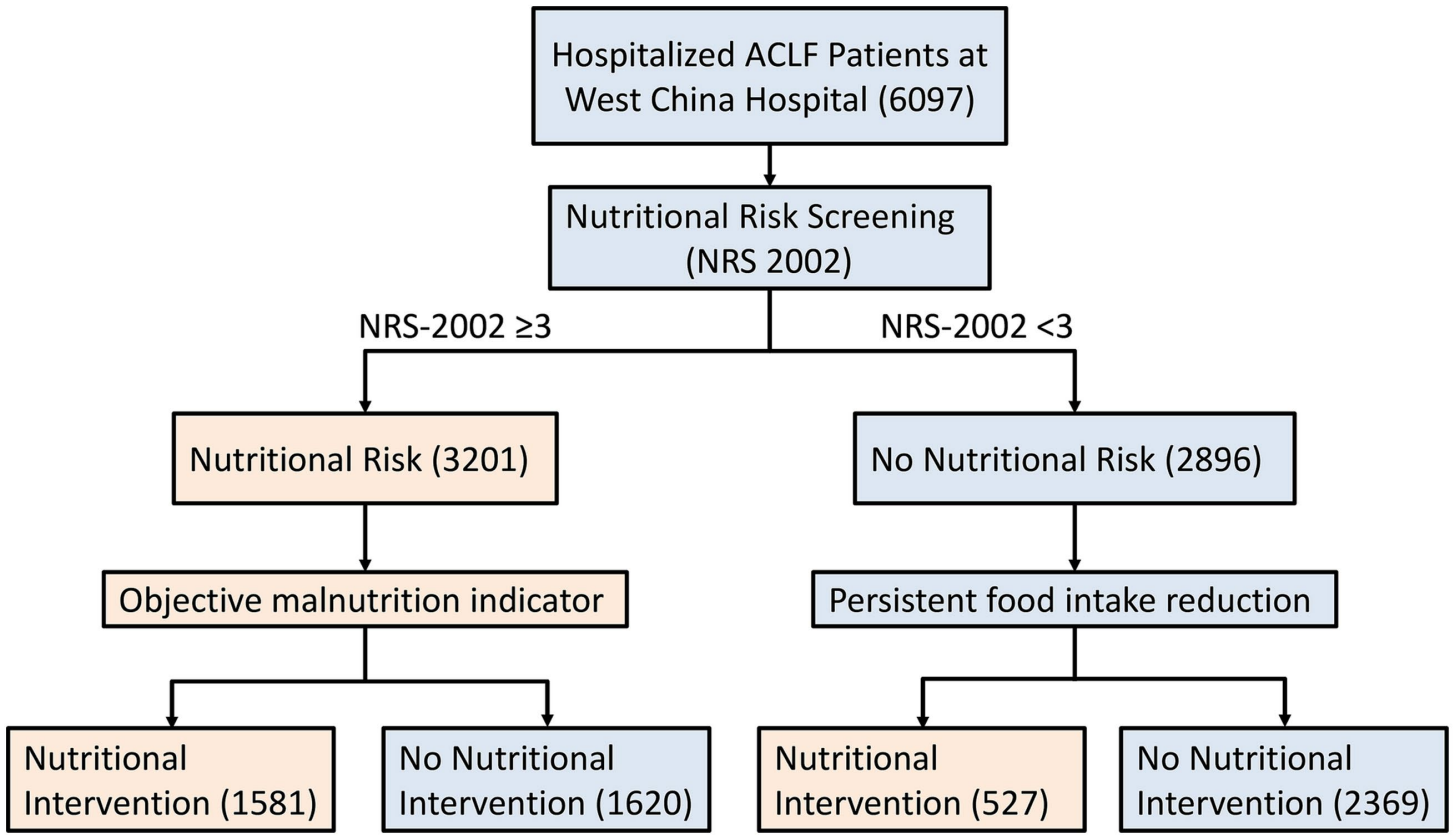
Patient enrollment flow diagram.

A total of 6,097 ACLF patients were included in this study, with a flow diagram shown in Figure 1. The median age was 53 years [interquartile range (IQR) 43, 64], and 69.2% of them were male. The proportion of patients with cirrhosis was 19.6% (1197). 555 (9.1%) were diagnosed with ascites, 733 (12.0%) with spontaneous bacterial peritonitis (SBP), 792 (13.0%) with infection, 191 (3.1%) with hepatorenal syndrome, 346 (5.7%) with hepatic encephalopathy (HE), 577 (9.46%) with coagulation disorder, 290 (4.8%) with variceal bleeding. Infection was defined as systemic bacterial or viral infections confirmed by positive microbiological cultures or clinical evidence. SBP was classified as a separate, ACLF-specific complication, defined as bacterial infection of ascitic fluid. Among all patients, 171 (2.8%) died and 3,201 (52.5%) patients were at nutritional risk. The results showed that patients with nutritional risk had a higher median age and a lower BMI (*p* < 0.001). Levels of aspartate transaminase (AST), total bilirubin (TBIL), and international normalized ratio (INR) were significantly higher in the nutritional risk group, while albumin levels were lower (*p* < 0.001). In terms of renal function, the nutritional risk group had higher creatinine levels and slightly lower estimated glomerular filtration rate (eGFR) (*p* < 0.001). Moreover, patients with nutritional risk had lower platelet and hemoglobin levels (*p* < 0.001). Regarding disease severity, patients with nutritional risk had higher Child–Pugh scores and longer hospital stays (*p* < 0.001), and significantly higher incidences of complications such as hepatorenal syndrome, HE, infection, and coagulation disorders (*p* < 0.01). Mortality was also higher in the nutritional risk group (*p* < 0.001) (Table 1).

**Table 1:** Clinical features of patients with and without nutrition risk.

Variables total (*n* = 6,097)		Nutrition risk		
		No (*n* = 2,896)	Yes (*n* = 3,201)	*p*
Age (years)	53.1 (43.1, 63.7)	51.8 (41.8, 60.7)	54.2 (44.7, 66.1)	<0.001
BMI (kg/m^2^)	23.1 (20.7, 25.6)	23.5 (21.5, 25.9)	22.5 (19.6, 25.2)	<0.001
Gender, *n* (%)				0.52
Male	4,220 (69.2)	2016 (69.6)	2,204 (68.8)	
Female	1877 (30.8)	880 (30.4)	997 (31.2)	
Smoking, *n* (%)				0.01
No	3,752 (72.7)	1722 (71.1)	2030 (74.1)	
Yes	1,410 (27.3)	702 (28.9)	708 (25.9)	
Drinking, *n* (%)				<0.001
No	3,719 (73.9)	1,693 (71.7)	2026 (75.9)	
Yes	1,313 (26.1)	670 (28.3)	643 (24.1)	
ALT (U/L)	77 (42, 96)	76 (42, 96)	78 (42, 96)	0.71
AST (U/L)	81 (53, 97)	77 (46, 96)	84 (59, 98)	<0.001
TBIL (μmol/L)	77.30 (8.7, 98.3)	72.4 (7.7, 97.5)	79.5 (9.3, 98.8)	<0.001
TBA (μmol/L)	67.4 (8.3, 205.6)	73.3 (8.0, 229.6)	58.9 (8.4, 158.1)	0.009
INR	1.59 (1.19, 2.19)	1.48 (1.10, 2.04)	1.69 (1.27, 2.35)	<0.001
Alb (g/L)	27.7 (24.4, 31.9)	28.8 (25.5, 34.4)	26.9 (23.2, 30.0)	<0.001
Creatinine (μmol/L)	80 (65, 95)	77 (64, 92)	84 (66, 96)	<0.001
eGFR (mL/min/1.73m^2^)	101.2 (64.3, 107.9)	101.8 (78.3, 108.9)	100.9 (48.6, 107.1)	<0.001
PLT (×10^9^/L)	108 (65, 152)	111 (73, 161)	107 (60, 142)	<0.001
Hb (g/L)	102 (93, 113)	105 (100, 121)	101 (76, 108)	<0.001
NRS2002	3 (1, 4)	1 (1, 2)	4 (3, 4)	<0.001
Child–Pugh score	8 (7, 9)	8 (6, 9)	9 (7, 10)	<0.001
Duration of hospital stay (days)	14 (8, 23)	13 (8, 21)	15 (9, 25)	<0.001
Nutrition intervention, *n* (%)				<0.001
No	3,989 (65.4)	2,369 (81.8)	1,620 (50.6)	
Yes	2,108 (34.6)	527 (18.2)	1,581 (49.4)	
Hypertension, *n* (%)				0.75
No	5,924 (97.2)	2,816 (97.2)	3,108 (97.1)	
Yes	173 (2.8)	80 (2.8)	93 (2.9)	
Diabetes, *n* (%)				0.74
No	5,875 (96.4)	2,793 (96.4)	3,082 (96.3)	
Yes	222 (3.6)	103 (3.6)	119 (3.7)	
Cirrhosis, *n* (%)				0.54
No	4,900 (80.4)	2,318 (80.1)	2,582 (80.7)	
Yes	1,197 (19.6)	578 (19.9)	619 (19.3)	
Plasma transfusion, *n* (%)				<0.001
No	2,920 (47.9)	1,596 (55.1)	1,324 (41.4)	
Yes	3,176 (52.1)	1,299 (44.9)	1877 (58.6)	
Edema, *n* (%)				<0.001
No	4,630 (75.9)	2,260 (78.1)	2,370 (74.1)	
Yes	1,467 (24.1)	636 (21.9)	831 (25.9)	
Ascites, *n* (%)				0.46
No	5,542 (90.9)	2,624 (90.6)	2,918 (91.2)	
Yes	555 (9.1)	272 (9.4)	283 (8.8)	
Variceal bleeding, *n* (%)				<0.001
No	5,807 (95.24)	2,805 (96.86)	3,002 (93.78)	
Yes	290 (4.76)	91 (3.14)	199 (6.22)	
Hepatorenal syndrome, *n* (%)				<0.001
No	5,906 (96.9)	2,836 (97.9)	3,070 (95.9)	
Yes	191 (3.1)	60 (2.1)	131 (4.1)	
Hepatic encephalopathy, *n* (%)				<0.001
No	5,751 (94.3)	2,769 (95.6)	2,982 (93.2)	
Yes	346 (5.7)	127 (4.4)	219 (6.8)	
Infection, *n* (%)				<0.001
No	5,305 (87.0)	2,572 (88.8)	2,733 (85.4)	
Yes	792 (13.0)	324 (11.2)	468 (14.6)	
Coagulation disorder, *n* (%)				0.002
No	5,520 (90.5)	2,657 (91.7)	2,863 (89.4)	
Yes	577 (9.5)	239 (8.3)	338 (10.6)	
Spontaneous bacterial peritonitis, *n* (%)				0.93
No	5,364 (88.0)	2,549 (88.0)	2,815 (87.9)	
Yes	733 (12.0)	347 (12.0)	386 (12.1)	
Death, *n* (%)				<0.001
No	5,926 (97.2)	2,856 (98.6)	3,070 (95.9)	
Yes	171 (2.8)	40 (1.4)	131 (4.1)	

BMI, body mass index; ALT, alanine transaminase; AST, aspartate transaminase; TBIL, total bilirubin; TBA, total bile acid; INR, international normalized ratio; Alb, albumin; eGFR, estimated glomerular filtration rate; PLT, platelet count; Hb, hemoglobin; NRS2002, Nutritional Risk Screening 2002; Child–Pugh score, Child–Pugh liver function classification score.

### Association of adjuvant nutritional support with the risk of clinical complications and all-cause mortality in patients with or without nutritional risk

Next, we analyzed the relationship between nutritional intervention and complications and all-cause mortality in ACLF patients with or nutritional risk using logistic regression.

**Figure 2:**
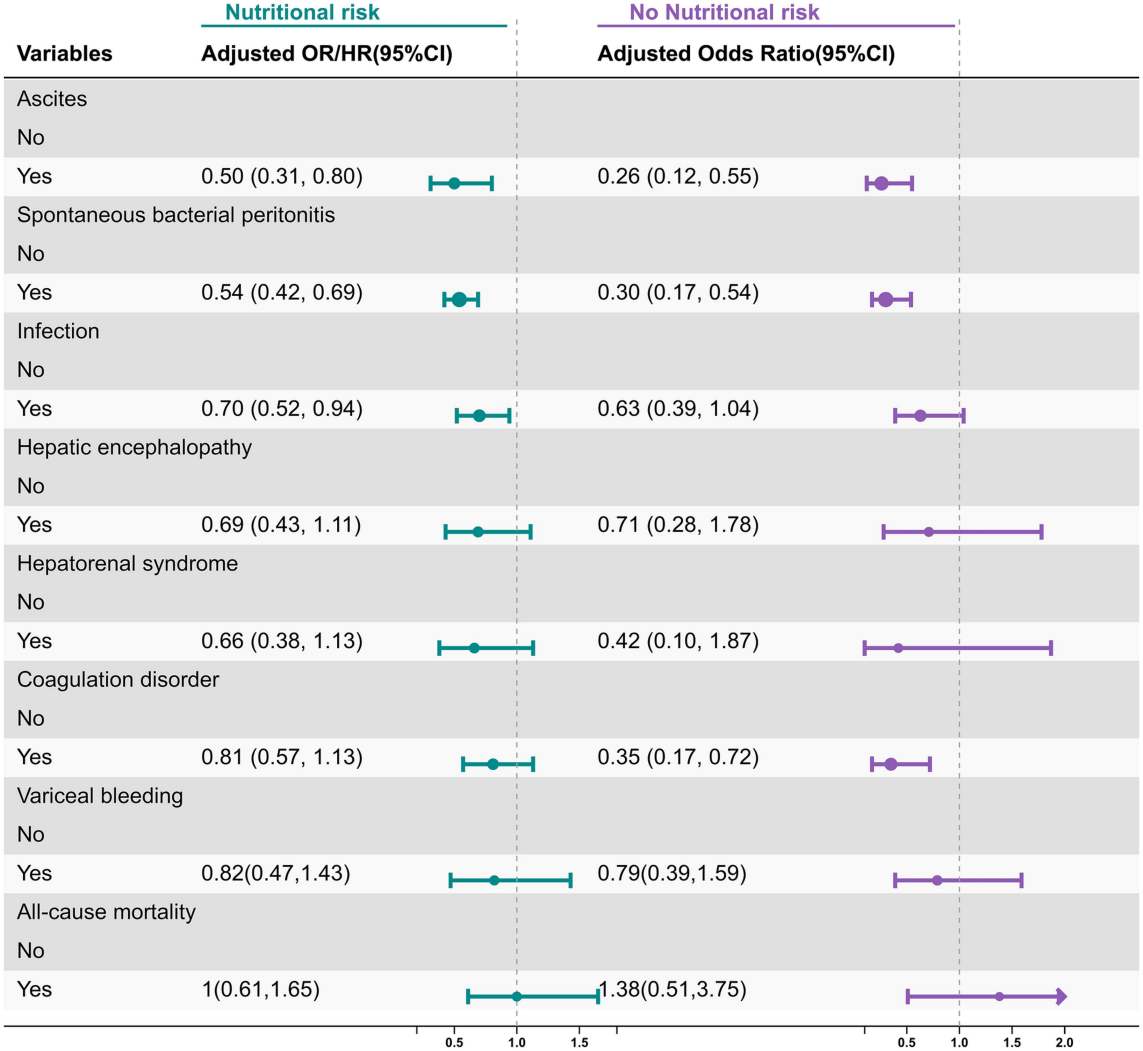
Association of adjuvant nutritional support with the frequency of clinical complications and all-cause mortality in patients with and without nutritional risk after multivariate logistic regression model.

After adjusting for age, gender, height, weight, smoking, drinking, hypertension, diabetes, and Child–Pugh score, we found that patients with nutritional risk receiving nutritional intervention were associated with lower frequency of ascites, SBP, and infection, with OR and 95% CI of 0.50 (0.31–0.80), 0.34 (0.24–0.49), and 0.70 (0.52–0.94), respectively. However, nutritional intervention was not associated with lower frequency of hepatorenal syndrome, hepatic encephalopathy (HE), coagulation abnormalities, or variceal bleeding risk with OR and 95% CI of 0.66 (0.38–1.13), 0.69 (0.43–1.11), 0.81 (0.57–1.13), and 0.82 (0.47–1.43) respectively (Figure 2). Cox regression was used to analyze the impact of nutritional intervention on all-cause mortality in patients with ACLF. After adjusting for age, gender, height, weight, smoking, drinking, hypertension, diabetes, and Child–Pugh score, we found that nutritional intervention had no association with all-cause mortality, with a hazard ratio (HR) and 95% CI of 1.00 (0.61–1.65) (Figure 2).

We further analyzed whether nutritional intervention was associated with lower frequency of clinical complications and all-cause mortality in patients without nutritional risk. We found that nutritional intervention was associated with lower frequency of ascites, SBP, and coagulation disorders, with OR and 95% CI of 0.26 (0.12–0.55), 0.30 (0.17–0.54), and 0.35 (0.17–0.72), respectively. However, it was not associated with lower frequency of infection, hepatorenal syndrome, HE, or variceal bleeding with OR and 95% CI of 0.63 (0.39–1.04), 0.42 (0.10–1.87), 0.71 (0.28–1.78), and 0.79 (0.39–1.59), respectively (Figure 2). Cox proportional hazards model showed that nutritional intervention had no significant effect on reducing all-cause mortality, with HR and 95% CI of 1.38 (0.51–3.75) (Figure 2).

### Stratified analysis by nutritional risk status and intervention effect

**Figure 3:**
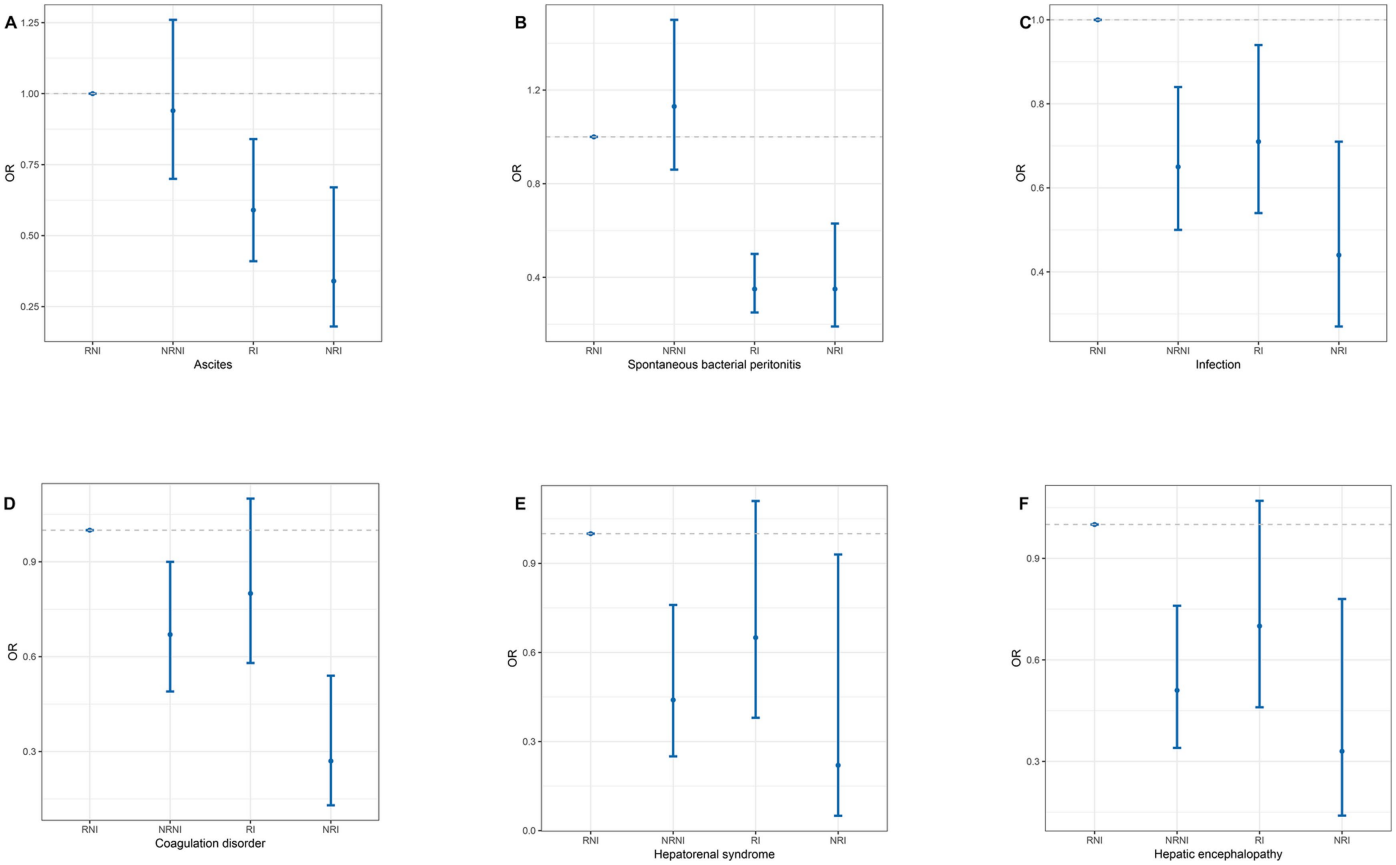
The interaction between nutritional risk and nutritional intervention in liver failure patients (panels **A–F**). Panels correspond to different clinical outcomes: **(A)** ascites, **(B)** spontaneous bacterial peritonitis, **(C)** infection, **(D)** coagulation disorder, **(E)** hepatorenal syndrome, **(F)** hepatic encephalopathy. RNI, nutritional risk without intervention; NRNI, no nutritional risk without intervention; RI, nutritional risk with intervention; NRI, no nutritional risk with intervention.

Moreover, we divided ACLF patients into four groups based on nutritional risk and nutritional intervention: nutritional risk without intervention, no nutritional risk without intervention, no nutritional risk with intervention, and nutritional risk with intervention. After multivariate regression analysis, patients with no nutritional risk with intervention was associated with lower frequency of ascites, SBP, and infection compared to those with nutritional risk without intervention, with OR and 95% CI being 0.34 (0.18–0.67), 0.35 (0.19–0.63), and 0.44 (0.27–0.71), while patients with nutritional risk with intervention also was associated with lower frequency of these outcomes compared to those with nutritional risk without intervention, with corresponding OR and 95% CI of 0.59 (0.41–0.84), 0.35 (0.25–0.50), and 0.71 (0.54–0.94). Additionally, patients with no nutritional risk without intervention was associated with lower frequency of infection than those with nutritional risk without intervention [0.65 (0.50–0.84)]. Moreover, after multivariate regression analysis, the group with no nutritional risk without intervention was associated with lower frequency of hepatorenal syndrome, HE, and coagulation disorders compared to those with nutritional risk without intervention, with OR and 95% CI of 0.44 (0.25–0.76), 0.51 (0.34–0.76), and 0.67 (0.49–0.90), respectively; meanwhile, the group with no nutritional risk with intervention also was associated with lower frequency of these complications compared to those with nutritional risk without intervention, with corresponding OR and 95% CI of 0.22 (0.05–0.93), 0.33 (0.14–0.78), and 0.27 (0.13–0.54) (Figure 3).

### Subgroups analysis by the administration route between parenteral nutrition and enteral nutrition

Among these intervened patients with nutritional risk, subgroup analysis by administration route confirmed consistent efficacy across all three modalities. EN, PN, and EN + PN all reduced the risks of ascites (EN: OR = 0.52, 95% CI: 0.33–0.82; PN: OR = 0.47, 95% CI: 0.28–0.79; EN + PN: OR = 0.49, 95% CI: 0.30–0.81), SBP (EN: OR = 0.36, 95% CI: 0.25–0.52; PN: OR = 0.31, 95% CI: 0.20–0.48; EN + PN: OR = 0.33, 95% CI: 0.19–0.58), and infection (EN: OR = 0.73, 95% CI: 0.54–0.99; PN: OR = 0.68, 95% CI: 0.47–0.98; EN + PN: OR = 0.70, 95% CI: 0.48–1.02), with consistent but non-significant effect sizes for hepatorenal syndrome, HE, and gastrointestinal bleeding.

Among the patients without nutritional risk who received intervention, subgroup analysis by administration route demonstrated consistent efficacy across modalities. EN, PN, and EN + PN all reduced ascites (EN: OR = 0.27, 95% CI: 0.12–0.59; PN: OR = 0.24, 95% CI: 0.09–0.63; EN + PN: OR = 0.25, 95% CI: 0.08–0.79), SBP (EN: OR = 0.31, 95% CI: 0.16–0.59; PN: OR = 0.28, 95% CI: 0.12–0.65; EN + PN: OR = 0.30, 95% CI: 0.11–0.82), and coagulation disorder (EN: OR = 0.37, 95% CI: 0.17–0.80; PN: OR = 0.32, 95% CI: 0.14–0.73; EN + PN: OR = 0.34, 95% CI: 0.12–0.96), with consistent direction of effect (though non-significant) for infection, hepatorenal syndrome, HE, and gastrointestinal bleeding across all three administration routes.

### Stratified analysis based on baseline characteristics

Moreover, we conducted subgroup analyses in patients with nutritional risk and found that the association between nutritional intervention and reduced risk of complications in patients with ACLF was not consistent in some subgroups. But the association of nutritional intervention and reduced risk of ascites and spontaneous bacterial peritonitis were robust in most subgroups (Table 2).

**Table 2:** Subgroup analysis of the association of adjuvant nutritional support with complication risk in patients with nutritional risk.

Variables	Ascites	SBP	Infection	Coagulation disorder	Hepatorenal syndrome	Hepatic encephalopathy	Variceal bleeding
	OR (95% CI)	OR (95% CI)	OR (95% CI)	OR (95% CI)	OR (95% CI)	OR (95% CI)	OR (95% CI)
Gender							
Male	0.57 (0.34, 0.98)	0.34 (0.23, 0.53)	0.74 (0.51, 1.06)	0.91 (0.59, 1.39)	0.93 (0.50, 1.74)	0.79 (0.44, 1.41)	0.73 (0.53, 1.02)
Female	0.28 (0.10, 0.78)	0.32 (0.16, 0.67)	0.60 (0.35, 1.03)	0.63 (0.35, 1.12)	0.22 (0.06, 0.81)	0.56 (0.23, 1.33)	0.97 (0.51, 1.82)
BMI							
<25	0.42 (0.24, 0.75)	0.41 (0.27, 0.62)	0.77 (0.54, 1.10)	0.93 (0.63, 1.39)	0.85 (0.41, 1.73)	0.92 (0.52, 1.64)	0.85 (0.56, 1.29)
>25	0.54 (0.17, 1.74)	0.19 (0.08, 0.45)	0.55 (0.29, 1.03)	0.35 (0.16, 0.78)	0.44 (0.16, 1.21)	0.29 (0.10, 0.82)	0.57 (0.27, 1.18)
Age							
<50	0.62 (0.30, 1.27)	0.28 (0.16, 0.48)	0.67 (0.43, 1.06)	0.78 (0.46, 1.32)	1.16 (0.49, 2.76)	0.66 (0.30, 1.44)	0.90 (0.59, 1.39)
>50	0.44 (0.23, 0.84)	0.40 (0.24, 0.65)	0.72 (0.48, 1.08)	0.83 (0.53, 1.32)	0.44 (0.21, 0.91)	0.69 (0.38, 1.27)	0.73 (0.49, 1.08)
Cirrhosis							
No	0.41 (0.16, 1.04)	0.15 (0.06, 0.38)	0.91 (0.57, 1.46)	0.86 (0.50, 1.47)	1.45 (0.35, 5.94)	0.52 (0.24, 1.13)	0.63 (0.54, 1.76)
Yes	0.75 (0.41, 1.37)	0.56 (0.35, 0.90)	0.93 (0.59, 1.46)	1.20 (0.74, 1.93)	0.81 (0.43, 1.54)	1.13 (0.60, 2.11)	0.55 (0.27, 1.53)
Hypertension							
No	0.46 (0.28, 0.75)	0.31 (0.21, 0.45)	0.67 (0.49, 0.92)	0.75 (0.52, 1.07)	0.64 (0.35, 1.15)	0.72 (0.44, 1.20)	0.69 (0.50, 0.95)
Yes	2.72 (0.17, 43.80)	1.57 (0.36, 6.90)	2.10 (0.50, 8.90)	2.48 (0.57, 10.75)	1.42 (0.21, 9.77)	0.63 (0.11, 3.58)	2.13 (0.88, 5.18)
Diabetes							
No	0.42 (0.25, 0.70)	0.32 (0.21, 0.47)	0.63 (0.46, 0.87)	0.73 (0.51, 1.06)	0.63 (0.35, 1.15)	0.64 (0.39, 1.08)	0.80 (0.59, 1.09)
Yes	1.71 (0.39, 7.40)	0.56 (0.17, 1.88)	1.15 (0.38, 3.45)	1.39 (0.43, 4.48)	0.58 (0.10, 3.26)	1.10 (0.22, 5.44)	0.76 (0.31, 1.86)
Smoking							
No	0.47 (0.26, 0.85)	0.34 (0.21, 0.53)	0.53 (0.37, 0.77)	0.61 (0.40, 0.92)	0.39 (0.19, 0.83)	0.47 (0.25, 0.87)	0.72 (0.47, 1.08)
Yes	0.55 (0.24, 1.24)	0.36 (0.20, 0.64)	1.29 (0.76, 2.18)	1.50 (0.81, 2.80)	1.18 (0.50, 2.77)	1.21 (0.56, 2.61)	0.96 (0.58, 1.61)
Drinking							
No	0.46 (0.26, 0.83)	0.33 (0.20, 0.52)	0.71 (0.50, 1.02)	0.66 (0.44, 0.99)	0.38 (0.18, 0.79)	0.53 (0.29, 0.96)	0.67 (0.44, 1.01)
Yes	0.63 (0.28, 1.42)	0.38 (0.21, 0.69)	0.65 (0.37, 1.15)	1.35 (0.71, 2.55)	1.37 (0.56, 3.34)	1.11 (0.50, 2.48)	0.90 (0.53, 1.54)
Child–Pugh							
A	1.00 (0.00, Inf)	0.00 (0.00, Inf)	2.48 (0.36, 17.21)	1532897006.27 (0.00, Inf)	1.00 (0.00, Inf)	1.00 (0.00, Inf)	1.25 (0.18, 8.96)
B	1.20 (0.31, 4.70)	0.27 (0.14, 0.49)	0.71 (0.44, 1.14)	0.71 (0.42, 1.20)	0.94 (0.32, 2.75)	0.23 (0.06, 0.85)	0.86 (0.51, 1.47)
C	0.45 (0.27, 0.75)	0.43 (0.27, 0.67)	0.72 (0.48, 1.10)	0.87 (0.54, 1.39)	0.58 (0.30, 1.11)	0.91 (0.54, 1.52)	0.89 (0.62, 1.29)

SBP, spontaneous bacterial peritonitis; OR, odds ratio; CI, confidence interval.

In ACLF patients without nutritional risk, we conducted subgroup analyses and found that nutritional intervention was associated with lower frequency of ascites, SBP, and disorders in most subgroup populations (Table 3).

**Table 3:** Subgroup analysis of the association of adjuvant nutritional support with complication risk in patients without nutritional risk.

Variables	Ascites	SBP	Infection	Coagulation disorder	Hepatorenal syndrome	Hepatic encephalopathy	Variceal bleeding
	OR (95% CI)	OR (95% CI)	OR (95% CI)	OR (95% CI)	OR (95% CI)	OR (95% CI)	OR (95% CI)
Gender							
Male	0.29 (0.12, 0.69)	0.26 (0.13, 0.55)	0.63 (0.34, 1.16)	0.19 (0.07, 0.54)	0.00 (0.00, Inf)	0.89 (0.31, 2.59)	0.57 (0.27, 1.21)
Female	0.20 (0.04, 0.94)	0.37 (0.14, 1.02)	0.62 (0.26, 1.50)	1.05 (0.36, 3.00)	1.19 (0.23, 6.27)	0.26 (0.03, 2.14)	0.73 (0.21, 2.52)
BMI							
<25	0.20 (0.08, 0.50)	0.23 (0.11, 0.49)	0.62 (0.33, 1.14)	0.26 (0.10, 0.68)	0.54 (0.12, 2.49)	0.76 (0.24, 2.39)	0.67 (0.28, 1.61)
>25	0.30 (0.07, 1.20)	0.51 (0.20, 1.32)	0.66 (0.28, 1.58)	0.55 (0.18, 1.66)	0.00 (0.00, Inf)	0.62 (0.12, 3.07)	0.52 (0.12, 2.23)
Age							
<50	0.19 (0.06, 0.66)	0.20 (0.08, 0.53)	0.46 (0.20, 1.07)	0.23 (0.07, 0.77)	0.94 (0.11, 8.32)	0.58 (0.12, 2.90)	0.90 (0.38, 2.16)
>50	0.36 (0.14, 0.96)	0.45 (0.21, 0.96)	0.80 (0.43, 1.51)	0.48 (0.20, 1.19)	0.32 (0.04, 2.54)	0.79 (0.25, 2.53)	0.45 (0.18, 1.15)
Cirrhosis							
No	0.39 (0.09, 1.66)	0.43 (0.15, 1.21)	0.93 (0.45, 1.92)	0.59 (0.22, 1.60)	2.55 (0.34, 18.91)	0.57 (0.10, 3.33)	2.13 (0.71, 6.40)
Yes	0.80 (0.25, 2.52)	0.88 (0.34, 2.28)	1.37 (0.54, 3.48)	0.71 (0.22, 2.28)	0.00 (0.00, Inf)	2.16 (0.60, 7.79)	0.97 (0.40, 2.38)
Smoking							
No	0.15 (0.05, 0.44)	0.22 (0.10, 0.48)	0.49 (0.26, 0.90)	0.35 (0.16, 0.80)	0.58 (0.13, 2.64)	0.54 (0.15, 1.87)	0.53 (0.22, 1.25)
Yes	0.55 (0.18, 1.70)	0.53 (0.21, 1.34)	1.07 (0.45, 2.55)	0.35 (0.08, 1.56)	0.00 (0.00, Inf)	0.87 (0.20, 3.76)	0.44 (0.10, 1.91)
Drinking							
No	0.11 (0.03, 0.36)	0.19 (0.09, 0.44)	0.59 (0.32, 1.09)	0.46 (0.22, 0.97)	0.61 (0.13, 2.80)	0.94 (0.36, 2.43)	0.56 (0.23, 1.32)
Yes	0.74 (0.26, 2.10)	0.61 (0.25, 1.46)	0.74 (0.30, 1.81)	0.00 (0.00, Inf)	0.00 (0.00, Inf)	0.00 (0.00, Inf)	0.69 (0.20, 2.32)
Child–Pugh							
A	1.00 (0.00, Inf)	0.65 (0.00, Inf)	0.64 (0.07, 5.65)	0.87 (0.07, 10.52)	1.00 (0.00, Inf)	1.00 (0.00, Inf)	1.60 (0.17, 15.55)
B	0.28 (0.06, 1.28)	0.26 (0.11, 0.61)	0.73 (0.38, 1.41)	0.45 (0.18, 1.09)	0.41 (0.04, 3.87)	2.03 (0.63, 6.55)	1.51 (0.66, 3.45)
C	0.23 (0.10, 0.56)	0.33 (0.15, 0.73)	0.37 (0.16, 0.87)	0.15 (0.04, 0.66)	0.32 (0.04, 2.57)	0.15 (0.02, 1.19)	0.18 (0.04, 0.77)
Hypertension							
No	0.23 (0.11, 0.51)	0.30 (0.17, 0.55)	0.55 (0.32, 0.93)	0.28 (0.13, 0.63)	0.22 (0.03, 1.67)	0.73 (0.29, 1.84)	0.61 (0.31, 1.18)
Yes	41656966122033888.00 (0.00, Inf)	0.00 (0.00, Inf)	14.57 (0.62, 343.04)	4.70 (0.35, 63.33)	19.30 (0.20, 1827.72)	0.00 (0.00, Inf)	1.35 (0.14, 12.73)
Diabetes							
No	0.26 (0.12, 0.56)	0.28 (0.15, 0.51)	0.55 (0.33, 0.95)	0.37 (0.18, 0.76)	0.45 (0.10, 2.01)	0.72 (0.29, 1.82)	0.55 (0.27, 1.11)
Yes	0.00 (0.00, Inf)	2.10 (0.13, 34.08)	14133931.13 (0.00, Inf)	0.00 (0.00, Inf)	0.00 (0.00, Inf)	0.00 (0.00, Inf)	2.05 (0.38, 10.99)

SBP, spontaneous bacterial peritonitis; OR, odds ratio; CI, confidence interval.

## Discussion

The functional integrity of the liver is crucial for nutritional supply, and the liver plays a fundamental role in intermediate metabolism. Therefore, patients with liver disease often have nutritional risks. This study included 6,097 patients with ACLF, we found that for patients with nutritional risk, nutritional intervention could significantly reduce the risks of ascites, spontaneous bacterial peritonitis, and infection. In addition, for ACLF patients without nutritional risk, nutritional intervention could also significantly reduce the risks of ascites, spontaneous bacterial peritonitis, and coagulation abnormalities. However, nutritional intervention had no obvious effect on reducing the all-cause mortality of ACLF patients.

Liver failure leads to a hypermetabolic state and an increase in resting energy expenditure (REE). It is estimated that REE increases by 18–30% (17). However, energy production capacity decreases due to changes in mitochondrial structural proteins and ATP depletion caused by oxidative stress in liver failure (20). Extensive hepatocyte damage reduces energy utilization efficiency (12, 14). In addition, patients with liver failure also have energy metabolism disorders. Hepatic glycogen depletion, impaired gluconeogenesis due to hepatocyte dysfunction, and insulinemia caused by increased insulin secretion and decreased degradation make patients prone to hypoglycemia, which can promote the occurrence of hepatic encephalopathy (1, 5, 12). Liver failure can also cause hyperglycemia due to insulin resistance and glucagonemia (5). Insulin resistance occurs early in liver failure, promoting a catabolic state and leading to muscle atrophy and myasthenia (18). Due to amino acid leakage caused by hepatocyte necrosis, reduced amino acid utilization, and amino acid release during systemic protein catabolism, patients with liver failure usually have elevated blood ammonia levels (21). In addition, the protein synthesis capacity of the liver also decreases. Therefore, the protein requirement of patients with liver failure increases. As the glucose utilization efficiency of patients with liver failure decreases, endogenous lipid utilization increases accordingly. However, lipoprotein metabolism is also impaired due to reduced synthesis of proteins, fatty acids, and cholesterol (22). Lipoprotein metabolism disorders in patients with liver failure lead to an increase in cholesterol content in the cell membrane, which weakens cell deformability by reducing membrane fluidity and makes cells more vulnerable (20). Thus, the metabolic disorders in patients with liver failure are characterized by “high consumption, low synthesis, and multiple imbalances.” Increased energy expenditure and interference with the metabolism of essential nutrients mean that patients with liver failure often face nutritional risks.

In our study, we found that 52.5% of liver failure patients were at nutritional risk. The prevalence of malnutrition in patients with cirrhosis ranges from 5 to 92% due to differences in screening methods and study populations (23). In our study, the proportion of cirrhotic patients was only 19.6%, indicating that many liver failure patients without a cirrhotic basis also have nutritional risks. Patients with liver failure also tend to have hypermetabolism (24). Hypermetabolism is a cause of malnutrition in patients with end-stage liver disease.

In addition, our study found that nutritional support can reduce the incidence of ascites in patients with liver failure, regardless of nutritional risk. Study revealed that in patients with liver cirrhosis, sarcopenia is associated with an increased cumulative incidence of ascites (RR = 1.827, 95% CI 1.259–2.651) (25), and branched-chain amino acid supplements can reduce ascites and improve the quality of life in patients with liver cirrhosis (26). Liver failure often leads to hypoalbuminemia due to decreased hepatic protein synthesis. Nutritional support, particularly when supplemented with adequate protein and essential amino acids, can enhance hepatic protein synthesis and increase serum albumin levels. By restoring oncotic pressure, Nutritional intervention effectively reduces the accumulation of ascitic fluid. Proper nutritional management can also improve overall liver function. Moreover, ascites can increase the resting energy expenditure in patients with liver cirrhosis (27).

Our study also showed that adjuvant nutritional support can significantly reduce the occurrence of spontaneous bacterial peritonitis. Research indicated that sarcopenia is linked to a higher cumulative incidence of spontaneous bacterial peritonitis in cirrhotic patients (relative risk = 3.331, 95% CI 1.404–7.903) (25). Studies have also shown that low 25-hydroxyvitamin D levels are associated with spontaneous bacterial peritonitis in patients with liver cirrhosis (28). However, vitamin D supplementation can reduce infections and mortality in cirrhotic patients with spontaneous bacterial peritonitis (29). Adjuvant nutritional support can reduce the incidence of bacterial peritonitis by enhancing immune function through nutritional supplementation, maintaining intestinal barrier integrity to inhibit bacterial translocation, regulating metabolic balance and inflammatory responses, and improving the properties of ascitic fluid to suppress bacterial proliferation.

A key clinical question arises: which plays a more important role in ACLF outcomes—baseline nutritional risk or nutritional intervention? Our interaction analysis provides insights. Baseline nutritional risk is a strong prognostic determinant, while nutritional intervention is a potent modifiable factor that mitigates this risk. Compared to patients with NRNI, those with RNI had ~2.8–2.9-fold higher risks of ascites and SBP, highlighting the substantial impact of baseline nutritional risk. However, nutritional intervention reduced these excess risks by 30–65% in high-risk patients, and further lowered complications in low-risk patients. While the effect size of baseline nutritional risk is slightly larger than that of intervention, intervention’s ability to offset baseline risk and improve outcomes across all risk strata underscores its clinical utility. Importantly, the “no nutritional risk + intervention” group achieved the lowest complication rates, indicating that neither factor is mutually exclusive. Baseline risk identifies high-priority patients for screening, while intervention delivers tangible benefits regardless of risk status. This finding reinforces clinical priorities. Routine nutritional risk screening and universal personalized intervention, rather than prioritizing one factor over the other.

Moreover, a notable finding is that adjuvant nutritional support significantly reduces key complications in ACLF patients but does not improve all-cause mortality. This discrepancy reflects the unique pathophysiology of ACLF and the role of nutritional intervention. First, ACLF mortality is primarily driven by irreversible multi-organ failure and severe hepatocellular damage (2, 3), processes that nutritional support cannot reverse. Even with fewer complications, advanced organ dysfunction remains fatal (2). Second, baseline disease severity offsets complication-related benefits. Patients with nutritional risk have higher Child–Pugh scores and pre-existing organ impairment (8), limiting survival gains despite fewer complications. Third, nutritional intervention acts as a supportive rather than curative measure (12). It optimizes metabolism and reduces decompensation but cannot promote hepatic regeneration or resolve systemic inflammation, the root causes of ACLF mortality (3, 14). Finally, selection bias in the non-intervention group may dilute potential survival differences (11). This aligns with prior studies (11) showing nutritional support improves complication rates but not mortality in severe liver failure. Clinically, its value lies in reducing invasive interventions and improving quality of life, buying time for transplant evaluation or hepatic recovery (2, 12).

In conclusion, this study investigated through a large-sample clinical retrospective analysis that nutritional interventions can reduce complications in patients with liver failure. However, this study also has limitations. First, as a retrospective observational study, the non-intervention group was not randomly assigned but consisted of highly selected ACLF patients (mild disease, terminal multi-organ failure, or patient refusal), leading to potential selection bias. Although we adjusted for key confounders (age, comorbidities, disease severity) and performed severity-stratified analyses, residual confounding from unmeasured factors cannot be excluded, and causal inferences cannot be drawn. Randomized controlled trials are needed to confirm the causal relationship between nutritional intervention and reduced complications. Second, the proportion of nutritional risk may differ between liver failure caused by alcoholic liver disease and metabolic liver disease, but this study could not distinguish these patients within the liver failure cohort. Third, the study lacked anthropometric indicators and serial nutritional biomarkers for comprehensive nutritional assessment. Fourth, outcome measures were limited to acute clinical complications and all-cause mortality, lacking patient-centered outcomes and liver function recovery metrics. While acute complications are critical drivers of ACLF mortality, patient-reported outcomes and nutritional/hepatic recovery indicators would better reflect the holistic benefits of nutritional intervention. This omission is due to retrospective data collection constraints, and future prospective studies should adopt a multidimensional outcome framework to enhance clinical relevance. Finally, the study lacked data on long-term outcomes, which limits our understanding of the sustained effects of nutritional intervention.

## Conclusion

This retrospective study found that over half of liver failure patients have nutritional risk. Adjuvant nutritional support was associated with lower frequency of ascites, infection, and spontaneous bacterial peritonitis. Notably, even among patients without nutritional risk, nutritional intervention was associated with lower frequency of ascites, spontaneous bacterial peritonitis, and coagulation disorders. Our study highlighted the importance of routine nutritional screening and personalized nutritional interventions in clinical practice.

## Data Availability

The original contributions presented in the study are included in the article/supplementary material, further inquiries can be directed to the corresponding author.

## Footnotes

1
www.zstats.net
